# The role of telomeres in the mechanisms and evolution of life-history trade-offs and ageing

**DOI:** 10.1098/rstb.2016.0452

**Published:** 2018-01-15

**Authors:** Andrew J. Young

**Affiliations:** School of Biosciences, University of Exeter Penryn Campus, Penryn TR10 9FE, UK

**Keywords:** telomere, senescence, oxidative stress, cancer, constraint, adaptation

## Abstract

Evolutionary biology and biomedicine have seen a surge of recent interest in the possibility that telomeres play a role in life-history trade-offs and ageing. Here, I evaluate alternative hypotheses for the role of telomeres in the mechanisms and evolution of life-history trade-offs and ageing, and highlight outstanding challenges. First, while recent findings underscore the possibility of a proximate causal role for telomeres in current–future trade-offs and ageing, it is currently unclear (i) whether telomeres ever play a causal role in either and (ii) whether any causal role for telomeres arises via shortening or length-independent mechanisms. Second, I consider why, if telomeres do play a proximate causal role, selection has not decoupled such a telomere-mediated trade-off between current and future performance. Evidence suggests that evolutionary constraints have not rendered such decoupling impossible. Instead, a causal role for telomeres would more plausibly reflect an adaptive strategy, born of telomere maintenance costs and/or a function for telomere attrition (e.g. in countering cancer), the relative importance of which is currently unclear. Finally, I consider the potential for telomere biology to clarify the constraints at play in life-history evolution, and to explain the form of the current–future trade-offs and ageing trajectories that we observe today.

This article is part of the theme issue ‘Understanding diversity in telomere dynamics’.

## 1. The problem: the role of telomeres in life-history trade-offs and ageing

A core goal of evolutionary biology is to explain the remarkable diversity among organisms in the patterns of growth, reproduction and senescence that characterize life histories [1–16]. Examining variation in life-history traits, such as fecundity and lifespan, frequently reveals negative correlations between them, termed life-history trade-offs [1–3]. Such trade-offs can be apparent in comparisons among species (e.g. species with higher fecundity may have shorter lifespans [1–3]) and among individuals of the same species (e.g. individuals that invest more heavily in reproduction may age more quickly [17,18]). Life-history trade-offs have attracted particular interest in part because they pose a fundamental problem: why has selection not simultaneously maximized all life-history traits, leading to the evolution of an organism with infinite fecundity and lifespan; a so-called ‘Darwinian demon’ [19]? Explanations for the existence of life-history trade-offs hinge upon the invocation of evolutionary constraints (defined in their broadest sense as restrictions or limitations on the course or outcome of evolution [20]), which bound evolutionary potential such that all life-history traits are not simultaneously maximized [1,2,19,20]. Elucidating the nature of the evolutionary constraints at play in life-history evolution has, therefore, been a long-standing focus of mechanistic research in evolutionary biology [9–16,21,22]. Recent years have seen increasing congruence too with the research goals of biomedicine, where identification of the proximate mechanisms that underpin the life-history trade-offs that shape senescence holds the promise of interventions to alleviate natural limits on healthspan and lifespan. It is in this context that both evolutionary biology and biomedicine have seen a surge of recent interest in the role that telomeres may play in life-history trade-offs and senescence (hereafter ‘ageing’).

### (a) Life-history evolution and the search for underlying mechanisms

Discussions of the role that telomeres may play in the mechanisms and evolution of life-history trade-offs and ageing can be usefully grounded in life-history theory [1,2], whose logic is paralleled by that of the disposable soma theory of ageing ([4–6]; itself compatible with the antagonistic pleiotropy theory of ageing [5–7]). Life-history theory recognizes that the range of attainable evolutionary outcomes will be bounded by so-called ‘absolute’ evolutionary constraints (inescapable constraints born of the laws of physics), such as the need to allocate limited resources across multiple competing traits, including growth, reproduction and ‘somatic maintenance’ (the suite of molecular proof-reading and damage mitigation mechanisms that slow the progressive accumulation of errors and damage in the body's tissues [4–6]) [1,2,9–11,20]. Selection for optimal resource utilization strategies, for example, might then be expected to give rise to negative phenotypic and genetic correlations between some pairs of life-history traits (i.e. trade-offs; trade-offs need not be apparent between *all* pairs of traits as the optimal strategy may be to co-express a given pair while trading them off against others). This approach can intuitively account for the existence of trade-offs between traits that are simultaneously expressed, such as clutch size and egg size, as resource invested in one trait cannot be invested in others. And it can also account for ‘current–future’ life-history trade-offs, such as those between *current* reproduction or growth and *future* reproduction or survival. For example, current–future trade-offs are often envisaged to arise because current investment in reproduction can entail shortfalls in investment in somatic maintenance that will accelerate age-related declines in somatic integrity (the extent to which the body's tissues are free from errors and damage) that, unless recovered, are carried forward to future time steps, with detrimental effects on future reproduction and survival. In this way, current–future trade-offs have the potential to deflect an organism's senescence trajectory (characterized by the onset and rate of late-life declines in components of fitness), due to impacts of current actions on the rate of age-related decline in somatic integrity [8,17,18].

This resource allocation model has proved a useful heuristic for studying the evolution of life histories, but the extent to which the relevant evolutionary constraints in reality conform to the model's assumptions is currently far from clear [1–3,9–12,20–23]. While ‘absolute’ constraints of various kinds clearly will shape life-history evolution, the nature of the absolute constraints at play and their relative importance is less clear [1,2]. Moreover, the importance of what I shall collectively term ‘mechanistic constraints' (i.e. evolutionary constraints born of aspects of an organism's existing genetic, developmental and physiological mechanisms, themselves a product in part of phylogenetic history) in life-history evolution remains a matter for debate [9,12,20–23]. For these reasons, evolutionary biologists have become increasingly concerned with elucidating the mechanisms that underpin life-history trade-offs, with a view to shedding light on the constraints at play [9–16,21,22]. One major focus is the role that oxidative stress may play, given the potential for energetically demanding current activities to cause oxidative damage to diverse biomolecules, thereby compromising somatic integrity and future performance [13–15]. However, uncertainty regarding the central importance of oxidative stress [13–15], coupled with the expectation that multiple mechanisms of somatic deterioration are likely to act in concert [24,25], motivates continued attention to the roles of other pathways. Intuitively, in seeking a proximate mechanism that underpins current–future trade-offs one might seek a broadly conserved biological structure that can be damaged by ‘current’ actions, and whose deficits pass forward to ‘future’ time steps with the potential for causal detrimental effects on future performance. A wealth of evidence from biomedical and ecological research now highlights that telomeres may constitute just such a structure [16].

### (b) What role might telomeres play?

Telomeres are nucleoprotein complexes that cap the ends of the linear chromosomes of eukaryotes [26]. They comprise a repetitive non-coding DNA sequence (TTAGGG repeats in vertebrates) bound up in a multi-protein complex [26]. Their structural conservation across eukaryotes suggests an ancient origin and reflects their importance in overcoming two challenges posed by the evolution of linear chromosomes [26,27]. First, as conventional DNA polymerases do not replicate the ends of linear chromosomes, the presence of terminal non-coding telomeric DNA averts the loss of coding nucleotides during cell replication [28,29]. Second, the telomeric-binding proteins wrap up the chromosomal end in such a way as to shroud it from detection by the cell's DNA repair machinery, to avoid triggering a DNA damage response and/or pathological chromosomal fusions [26,30]. Telomeres are, however, dynamic structures that can change in length, and it is these telomere length dynamics, born of the balance of telomere attrition (the shortening of telomeres over time via the loss of terminal repeats) and elongation (e.g. the enzyme telomerase can extend telomeres via the addition of terminal telomeric repeats [26,31]), that are of particular interest when considering their role in the mechanisms and evolution of life histories.

Interest in a potentially causal role for telomere length dynamics in current–future trade-offs and ageing stems from a series of findings that highlight their potential to leave current actions having negative downstream effects on future performance [16]. First, while telomerase expression in unicellular eukaryotes is thought to maintain telomere lengths within a species-specific range, telomerase insufficiency or complete repression is commonplace in the somatic tissues of metazoans, frequently leading to progressive declines in telomere length with advancing organismal age [26,27,31–34]. Second, *in vitro* studies suggest that such shortening arises principally from cellular replication and oxidative stress [28,29], providing candidate pathways by which organismal growth and reproduction could accelerate telomere shortening [16]. Third, the accrual of short telomeres within cells is implicated in the triggering of apoptosis (programmed cell death) and a state of irreversible cell-cycle arrest termed cellular senescence; cell fates that are strongly implicated in age-related declines in tissue and organismal performance [25,35]. Accordingly, shorter mean telomere lengths *in vivo* frequently predict poorer organismal health and survival outcomes both in the laboratory and the wild [36]. As such, telomere attrition is now widely recognized as one of the hallmarks of ageing [25], and telomeric assessments are being widely adopted in evolutionary biology as a biomarker of somatic integrity. Despite this, limited attention has been paid to addressing the fundamental and increasingly pressing question posed by these relationships: what role, if any, do telomeres play in the mechanisms and evolution of life-history trade-offs and ageing?

Here, I seek to address this question by evaluating the mechanistic and evolutionary plausibility of telomeres playing a proximate causal role in current–future life-history trade-offs (and their impacts on ageing trajectories), before considering the wider question of whether telomere biology is likely to shed new light on the evolution of life-history trade-offs and ageing. First, I consider the case for telomeres being one of the proximate causal mechanisms that gives rise to current–future trade-offs (§2). I conclude that while it is mechanistically plausible that telomeres are one proximate cause of current–future trade-offs in a subset of species, whether telomere attrition *per se* plays a significant causal role in any species, relative to alternative mechanisms, is currently far from clear.

Second, I consider the evolutionary plausibility of telomeres being one proximate cause of current–future trade-offs (§3), given that selection would be expected to decouple such mechanistic links between current and future performance in the absence of evolutionary constraint (see above). In evaluating adaptive explanations for a strategy of progressive telomere attrition in somatic cells, I consider two broad forms of explanation that are not mutually exclusive: the *costly maintenance hypothesis* (telomere attrition is adaptive because telomere maintenance entails costs, such as the utilization of resources that could be invested in other traits) and the *functional attrition hypothesis* (telomere attrition is adaptive because it serves a particular *function*; such as its widely invoked function in countering cancer). I also highlight a potential and hitherto unexplored signalling function of telomere attrition: as a biological clock for regulating the pace of an individual's life history according to somatic integrity (the *life-history regulation hypothesis*).

Finally, I consider the extent to which telomere biology is likely to shed new light on the evolution of life-history trade-offs and ageing (§4). Importantly, evidence of a proximate causal role for telomeres in current–future trade-offs and ageing would not implicate a role for telomeres in shaping their evolution. Whether telomere biology informs our understanding of the evolution of life histories and ageing trajectories hinges upon (i) the extent to which telomere biology sheds new light on the constraints that explain why selection has not decoupled current–future trade-offs (see above) and (ii) the extent to which the evolution of telomeric mechanisms has influenced the ‘form’ of current–future trade-offs (i.e. the probability, magnitude and timing of the impacts of current actions on future performance).

## 2. Telomere attrition as a proximate cause of life-history trade-offs and ageing

Here I consider the mechanistic plausibility of a proximate causal role for telomeres in current–future trade-offs and ageing. I start by briefly considering the evidence that current reproduction and growth can accelerate telomere attrition, and that telomere attrition in turn can have causal negative effects on future organismal performance. Notably, the vast majority of cellular and organismal research in these areas has focused on vertebrates, in particular mammals and birds, and so the relevance of these patterns for other eukaryotic groups cannot be assumed (e.g. [37]). I then highlight key reasons for caution regarding the case for causality, including the possibility that telomere attrition acts as a non-causal biomarker of declines in somatic integrity, the likely importance of alternative mechanisms (including a causal role for telomeres independent of their length) and the limited taxonomic generality of a causal role for telomeres. The evolutionary plausibility of telomere attrition acting as a proximate cause of current–future trade-offs and ageing is addressed in the next section (§3).

### (a) Do current reproduction, growth and adversity accelerate telomere attrition?

Telomere attrition can arise through a range of processes [28,29], but two principal mechanisms provide candidate pathways by which current reproduction and growth could hasten telomere attrition [16,38]. First, in the absence of telomerase, telomeres shorten during each round of cellular replication, due in part to the inability of conventional DNA polymerases to completely replicate the ends of linear chromosomes [28,29]. Second, larger tracts of telomeric repeats are occasionally lost in seemingly ‘sporadic’ events [39], which appear to be attributable in part to oxidative damage to telomeric DNA ([28,29], but see [40]). Both cellular replication and oxidative damage provide viable mechanisms by which organismal growth and reproduction could impact telomere attrition. Growth and reproduction typically both entail significant cellular replication, and investments in either are generally expected to increase exposure to oxidative damage via associated increases in the rates of reactive oxygen species (ROS) production and/or compromised investment in antioxidant defences [13,14,38]. Accordingly, telomere attrition rates *in vivo* are often faster during the growth phase than later in life (e.g. [34,38]), and evidence suggests that growth acceleration may indeed entail oxidative stress and accelerate telomere shortening ([41,42], see [38] for a review). Whether reproduction entails an oxidative stress cost remains a matter of debate, as a recent meta-analysis has revealed that, while breeding females exerting greater reproductive effort show higher levels of oxidative damage, breeders typically show lower levels of oxidative damage than non-breeders [14]. These patterns have led to the suggestion that selection has favoured physiological strategies that mitigate oxidative stress during reproductive episodes (the oxidative shielding hypothesis [14]). Nevertheless, despite limited empirical attention, a number of studies have now provided evidence consistent with the expectation that reproductive effort accelerates telomere attrition (e.g. [43–45]). The proximate mechanisms that generate current–future trade-offs may also underpin the widely documented deleterious effects of current-life adversity on future performance [46,47]. It is notable then that exposure to diverse stressors as well as elevated glucocorticoid (stress hormone) concentrations has been demonstrated to accelerate telomere attrition, *in ovo*, *in utero*, during post-natal development and in adulthood (see [46,47] for recent reviews).

### (b) Does telomere attrition causally impact future organismal performance and ageing?

Telomere attrition could have causal negative effects on later-life performance because the accumulation of critically short telomeres in cells is understood to trigger apoptosis or cellular senescence; cell fates strongly implicated in organismal ageing [25,35,48–50]. The progressive loss of telomeric repeats is thought to gradually undermine the shielding of the chromosomal end from the cell's DNA damage recognition machinery [26,30], ultimately triggering a DNA damage response that in turn induces apoptosis or cellular senescence [26,30,35,50,51]. Accordingly, experimental evidence *in vitro* suggests that telomere attrition limits the proliferative potential of telomerase-negative cells, and the induction of telomerase expression restores proliferative potential and staves-off cellular senescence [51]. Caution is needed when interpreting the findings of telomerase manipulations, however, given the apparent potential for telomerase to rescue proliferative potential in the absence of telomere lengthening [26,52]. Moreover, recent findings have highlighted that while telomeric mechanisms may indeed be the major trigger of cellular senescence *in vivo* [53,54], such triggering may actually occur independent of telomere length [54,55] (see §2c(ii) below). Either way, the age-related accumulation of such senescent cells [53,54] *is* thought to play a key role in age-related declines in organismal performance, due to the associated passive loss of tissue function and their unusual pro-inflammatory secretory profiles [25,35]. Indeed, remarkable recent experiments have revealed that the clearance of senescent cells by two different methods counteracts age-related declines in performance in house mice, *Mus musculus* [48,49]. Telomere-attrition-induced apoptosis (as opposed to cellular senescence) is expected to compound these effects, as cell replacement via somatic stem cell division is generally expected to hasten stem cell telomere attrition and consequent stem cell exhaustion [25,35].

While it is widely accepted that telomere attrition can have causal effects on cell fates, the extent to which it contributes to age-related declines in *organismal* performance is less clear. Key areas of uncertainty include the relative importance of telomere-mediated and telomere-independent mechanisms (such as oxidative damage to other structures [13,35,50]), and the role of telomere-mediated mechanisms that may act independent of telomere length [54,55] (see next section). Nevertheless, given the panoply of mechanisms that could act in concert to precipitate organismal ageing [24,25], it is notable that three lines of evidence are at least consistent with a causal role for telomeres. First, humans and wild vertebrates with shorter mean blood cell telomere lengths and/or higher telomere attrition rates frequently show poorer survival prospects [36]. Moreover, recent comparative studies have revealed that the telomere attrition rates of wild animals are also higher in species with shorter lifespans and faster life histories [32,56]. While fewer studies have investigated whether individuals with shorter telomeres suffer poorer reproductive prospects, such relationships do exist (e.g. [57]). Second, short telomeres are also prognostic of age-related disease and poorer survival in humans [58], and telomeric pathologies are strongly implicated in diverse premature ageing disorders [25]. Third, studies revealing exacerbated tissue degeneration and shortened lifespans in telomerase-deficient house mice, and the restorative effects of telomerase restoration, illustrate the potential for telomere-related pathologies to causally impact organismal health (e.g. [59]). With regard to the causal role of telomeres in the natural ageing process (i.e. in the absence of telomerase deficiency), recent evidence that telomerase overexpression in adult and aged house mice extends natural lifespans is notable ([60], but see [61]). Indeed, there is considerable wider interest in the potential for telomerase manipulations to offer insights into the causal effects of changes in telomere length (see [62] for a review), despite the challenges posed by the diverse actions of telomerase aside from telomere lengthening [26,52].

### (c) Reasons for caution: the non-causal biomarker hypothesis, alternative mechanisms and questionable generality

Given cellular- and organismal-level evidence outlined above, it is certainly mechanistically *plausible* that telomere dynamics are one proximate cause of current–future trade-offs and ageing. However, multiple reasons exist for caution regarding causality. It is frequently highlighted, for example, that telomere length's utility as a predictor of health and fitness could instead reflect it acting as a non-causal biomarker of accumulated damage to other biological structures that themselves have causal deleterious effects on future performance (e.g. [61,63]; the *non-causal biomarker hypothesis*). There are also notable reasons for caution regarding the relative causal importance of telomere attrition and alternative mechanisms, as well as the taxonomic generality of any causal role for telomeres. I discuss these issues below.

#### (i) Telomeres as a non-causal ‘biomarker’: non-causation or weak causation?

Telomere lengths and/or attrition rates acting solely as non-causal biomarkers of accumulated damage to other biological structures could account not only for the prognostic utility of telomere dynamics (see above), but also for two findings that appear to discord with a simple mechanistic model in which some threshold telomere length triggers cellular senescence or apoptosis (though both patterns could also reflect the excessive simplicity of such a model [26,54,64]). First, observations that telomere attrition rate can be a stronger predictor of survival than telomere length (e.g. [34,45,63], but see [43] among others) could reflect telomere attrition rate correlating more closely with damage accumulation to other structures, perhaps because inter-individual variation in telomere length can arise via mechanisms other than damage accumulation [65,66]. Second, counter to expectation, recent meta-analyses have revealed that leucocyte telomere lengths are a stronger predictor of human survival in early than late life [58] (and, accordingly, population-level variation in human leucocyte telomere length does not appear to decline in late adulthood [61]). Given the marked effects of early-life adversity on telomere length (see above), this finding could reflect telomere length in early life acting as a biomarker of developmental stress, with this stress being the causal agent in early-life mortality (see [58,61] for discussion).

If telomere attrition played no causal role in current–future trade-offs and ageing, why then would telomeres be a useful biomarker of somatic integrity? One possibility is that telomere maintenance simply entails costs (see §3a), which leave it adaptive to tolerate telomere erosion arising via cellular replication and oxidative damage [28,29]; mechanisms that would then leave telomere length and attrition useful biomarkers of accumulated damage to other structures. Another potential explanation is that telomere length actually *functions* as a biomarker of somatic integrity, allowing the cell and/or organism to mount strategic responses accordingly (see §3b). For example, it is widely hypothesized that telomere attrition functions in part as a cancer surveillance mechanism, inducing senescence or apoptosis in cells that constitute a cancer risk [27,29,35,67] (see §3b(i)). Such a function need not necessarily be coupled with a causal role for telomere attrition in current–future trade-offs and ageing, as telomere attrition *per se* could trigger cellular senescence in cancers while actually playing little or no role in precipitating the accumulation of senescent cells in normal (non-cancerous) aged tissues ([54,55]; see §§2c(ii) and 3b(i)). In practice, it is likely to be difficult to tease apart a truly non-causal role for telomeres from a ‘weakly causal’ role in which telomere attrition is a minor player in a suite of causal pathways.

#### (ii) Alternative mechanisms could be of greater importance than telomere attrition

Telomere dynamics are clearly not the only mechanism that could causally link current actions to future performance, and whether telomere dynamics are major players in this suite of candidate mechanisms is currently far from clear. Most notably, oxidative damage alone has the potential to compromise tissue function both by simply accumulating in diverse biomolecules [13], and by triggering apoptosis and cellular senescence via telomere-independent mechanisms [35,50]. Indeed, such mechanistic redundancy may leave it challenging to detect a causal role for telomere attrition in current–future trade-offs and ageing. Experimental telomere elongation could, for example, alleviate telomere-induced apoptosis and cellular senescence only to have telomere-independent mechanisms trigger these same outcomes (but see [60]). Crucially, however, *in situ* hybridization studies have revealed that the majority of senescent cells in the skin of aged laboratory baboons, genus *Papio* [53], and house mice, *M. musculus* [54], show evidence of a persistent DNA damage response co-localized with telomeres, highlighting that telomeres could nevertheless be the primary trigger of cellular senescence *in vivo.*

Further complication arises, however, from recent evidence suggesting that the DNA damage response that triggers cellular senescence *in vivo* may be triggered *independent* of telomere length [54,55], not by the presence of critically short telomeres but by DNA damage located within telomeres (the repair of which is suppressed). Moreover, the findings suggest that such length-independent triggering of cellular senescence could be the primary route by which cellular senescence arises in normal (non-cancerous) aged tissues *in vivo* [54,55]*.* While such a mechanism could still implicate telomeres in the proximate causation of current–future trade-offs and ageing, the mechanism would be quite different to that widely envisaged. Indeed, such a telomere length-independent pathway may require the reinterpretation of many key findings previously attributed to effects of telomere length. Further investigation into the extent to which telomere attrition *per se* is relevant to the triggering of cellular senescence in normal-aged tissues *in vivo* should, therefore, be prioritized (see also §3b(i)).

#### (iii) The limited generality of a proximate causal role for telomeres

Telomeres are a eukaryotic phenomenon, showing broad conservation of structure across animals, plants, slime moulds, fungi, protozoa and algae [26,37]. While this taxonomic sweep is broad, it is notable that some prokaryotes, nevertheless, appear to age, due in part to the accumulation of oxidative damage [68,69]. This finding alone highlights the greater taxonomic generality of oxidative damage as a plausible causal agent of current–future trade-offs and ageing. Indeed, when viewed in this context, telomere dynamics may be neither a necessary nor a taxonomically sufficient proximate causal explanation for current–future trade-offs, though this does not of course preclude them acting as one causal mechanism in a subset of organisms.

A number of recent findings have also highlighted that progressive telomere shortening with age, which is central to the commonly invoked model of telomere-attrition-mediated trade-offs and ageing, may not be universal among vertebrates (see [37] for a review of the telomere biology of ectotherms). Longitudinal studies of several species have reported a lack of net telomere shortening in blood cells with advancing age, particularly in adulthood (e.g. [45,70]), and there is growing evidence suggestive of transient age-related *increases* in mean telomere length [71]. While these patterns could reflect a range of complications (including clonal turnover in stem cells stocks, autonomous proliferative dynamics and telomerase activity in lymphocytes and a degree of measurement error [33,71]), they, nevertheless, highlight the possibility that some species circumvent the late-life costs that could otherwise arise from progressive telomere attrition by actively maintaining their telomeres, perhaps via somatic telomerase expression in adulthood [37,72,73]. While humans show complete telomerase repression in most somatic cells in adulthood [31], there appears to be marked taxonomic variation in the extent of telomerase repression in adulthood (e.g. mammals [27,74], birds [73] and reptiles [37,72]). Comparative studies of telomerase expression in mammalian fibroblasts, for example, have revealed that the cells of smaller-bodied species show higher levels of telomerase expression [27,74]. While this pattern might lead one to suppose that smaller mammals may, therefore, circumvent telomere-attrition-mediated current–future trade-offs by maintaining their telomeres, the limited longitudinal data to date do not support this view. Most strikingly, while wild-type house mice show appreciable fibroblast telomerase expression *in vitro* (in contrast to telomerase repression in human fibroblasts; [27]), wild-type house mice, nevertheless, show extremely rapid telomere attrition rates *in vivo* (over 100 times the rates observed in humans [75]), and the prevalence and rate of accumulation of short telomeres both negatively predict residual lifespan [75]. Evidence of appreciable telomerase expression in somatic cells need not, therefore, undermine the potential for telomere attrition to play a causal role in current–future trade-offs and ageing. Indeed, recent comparative work offers an elegant solution to the otherwise paradoxical co-occurrence in the house mouse of appreciable somatic telomerase expression and very high telomere attrition rates: the latter could arise despite the former due to the weaker upstream DNA damage repair that may be typical of shorter-lived mammals [76].

## 3. Evolutionary explanations for telomeres acting as a proximate cause of life-history trade-offs and ageing

Suggestions that telomere attrition could be a proximate cause of current–future trade-offs and consequent variation in ageing trajectories are often met with scepticism regarding the evolutionary plausibility of this scenario. Why would selection not have decoupled a telomere-attrition-mediated causal mechanistic link between current and future performance (e.g. simply by favouring telomere length maintenance)? One conceivable explanation is that it actually would be adaptive to decouple such a link, but some form of evolutionary constraint born of an organism's existing genetic, developmental and physiological architecture (i.e. a mechanistic constraint) has rendered this impossible despite selection to do so. Several lines of evidence argue against this scenario. First, multiple mechanisms exist by which such decoupling might be achieved were it adaptive to do so: (i) maintenance of somatic cell telomere lengths via telomerase de-repression or alternative mechanisms (akin to the patterns observed in germ cells [26,27,31,77]); (ii) increasing the ‘initial’ telomere lengths of cell lineages (e.g. by modifying the set-points of telomere length homeostasis [26]); and (iii) the evolution of an alternative telomere structure (as has occurred in some plant and animal lineages [26,77]) that is more robust to oxidative damage or accessible for DNA repair [28,29]. Second, marked inter-specific, inter-individual and within-individual variation exists (often with heritable genetic components [78]) in the telomeric traits that selection might act upon to achieve such decoupling (e.g. telomere length [27,65,66,79] and telomerase expression [27,31,72,73,79]), highlighting their likely lability over developmental and evolutionary time. Indeed, recent comparative studies have highlighted evolutionary changes in both telomere length and telomerase expression consistent with adaptive explanations for attrition (e.g. [27,74]; see below). Collectively these patterns highlight no evident reason to suspect that a telomere-attrition-mediated mechanistic link between current and future performance (if one exists) has been maintained because mechanistic constraints have rendered its decoupling impossible. Instead it seems more likely that telomere attrition is an adaptive strategy, where it occurs, despite its potential for causing negative effects on later-life performance. I consider potential adaptive explanations below.

Adaptive explanations for telomere attrition hinge upon identifying fitness benefits arising from attrition that could offset any fitness costs arising from its downstream effects on performance. One might intuitively think that such fitness benefits would need to be substantial, if, for example, telomere attrition is the principal mechanism yielding current–future life-history trade-offs. However, very small fitness benefits might actually be sufficient if telomere attrition is only a minor causal player in current–future trade-offs relative to alternative mechanisms, particularly as any late-life fitness costs arising from attrition may be only weakly exposed to selection as few individuals survive to old age.

I suggest that it is worthwhile distinguishing two broadly different, but not mutually exclusive, adaptive explanations for telomere attrition, which I shall term the *costly maintenance hypothesis* and the *functional attrition hypothesis*. The costly maintenance hypothesis proposes that telomere attrition is adaptive simply because telomere length maintenance (e.g. via the prevention or repair of telomeric DNA damage and/or telomere elongation) entails costs which collectively render the toleration of attrition adaptive. Such costs could arise simply from the resources required for telomere maintenance (aligning this hypothesis with the logic of resource allocation approaches to life-history evolution; see introduction), but could also conceivably arise from other maintenance costs born of quirks of mechanism (see below). The functional attrition hypothesis, by contrast, proposes that telomere attrition is adaptive because attrition *per se* serves one or more beneficial *functions*. For example, one commonly invoked function for telomere attrition is that it acts as a cancer surveillance mechanism [29,35]. It is useful to distinguish these two forms of adaptive explanation as the costly maintenance hypothesis requires only that telomere maintenance entails costs (which is a certainty, regarding resource costs), while the functional attrition hypothesis requires that telomere attrition serves a beneficial function (which is less of a certainty; see below). I consider the plausibility of a role for each of these hypotheses below.

### (a) Telomere attrition as an adaptive strategy: the costly maintenance hypothesis

The costly maintenance hypothesis proposes that telomere attrition in the somatic cells of adults is adaptive because telomere length maintenance entails costs which collectively render tolerating attrition adaptive. Telomere maintenance must entail a resource cost of some kind, but it would seem implausible that this cost alone is so high as to account for selection favouring telomere attrition if attrition was a major causal agent in current–future trade-offs and ageing. Human telomeres, for example, have been estimated to shorten at a rate of just 20–30 bp per year, the restoration of which (including telomeric elongation and any associated changes in the shelterin complex, etc.) would presumably generate negligible resource demands relative to the maintenance and regulation of the 6.47 billion bp human genome [80]. The pervasive nature and quantity of so-called ‘junk’ DNA (see [81] for a recent review) and the success and persistence of polyploid genomes [82] also give cause to question whether the resource costs entailed in the maintenance of such a small genomic region (i.e. the telomeres) is likely to impact life-history evolution. That said, the potential importance of even small maintenance costs cannot currently be dismissed, as if telomere attrition is only a minor causal player in current–future trade-offs and ageing (see above) even small maintenance costs could conceivably be sufficient to favour the toleration of attrition.

The costs entailed in telomere maintenance need not arise solely from resource expense, however. It is conceivable that telomere maintenance entails other types of costs arising from interactions between biological mechanisms and the laws of physics. For example, aspects of telomere maintenance could conceivably entail risks to chromosomal integrity, and hence organismal fitness, if they require physical conformational rearrangements of the telomere (including its shelterin complex) or other chromosomal regions with which telomeres interact. While selection could have the potential to mitigate such costs via evolutionary changes to the mechanisms involved (e.g. the evolution of additional chromosomal stabilizing mechanisms), the costs associated with such solutions could conceivably be even higher than those that arise from telomere attrition. Alternatively, some form of absolute constraint might render the complete mitigation of such risks impossible. Closer attention to the plausibility of maintenance costs of this kind could be instructive, given their potential importance and the possibility that they cannot be readily reconciled with classical resource allocation models of life-history evolution.

As telomere maintenance must entail costs (born at least in part from resource expense), such costs alone could conceivably favour (i) telomere maintenance only in those cell lineages in which attrition would entail substantial fitness costs (e.g. germ cells and some immune cells [26,31]), while (ii) tolerating attrition in other somatic lineages, even if the latter had causal negative effects on later-life performance. The greater these causal negative effects are, however, the more acute the need becomes to invoke adaptive explanations for attrition born in part of a *function* for telomere attrition (i.e. a role for the functional attrition hypothesis).

### (b) Telomere attrition as an adaptive strategy: the functional attrition hypothesis

Telomere attrition in the somatic cells of adults could be adaptive (i.e. of net fitness benefit over the life-course), despite having causal negative effects of later-life performance, because progressive telomere attrition yields fitness benefits by serving one or more *functions* (the functional attrition hypothesis). While diverse functions are conceivable (e.g. [83]), I first consider the leading adaptive explanation for telomere attrition in the biomedical literature: that it serves to mitigate the risk and fitness consequences of cancer [29] (the *cancer surveillance hypothesis*). I then highlight the possibility that telomere attrition has an additional and hitherto unexplored function: allowing organisms to adaptively regulate their physiology, behaviour and life history according to residual somatic integrity (which I term the *life-history regulation hypothesis*).

#### (i) The cancer surveillance hypothesis

Cancer occurs, in part, when DNA mutations arise that overcome the mechanisms that tightly regulate cellular replication, leading to uncontrolled cellular proliferation with potentially catastrophic organismal consequences. The cancer surveillance hypothesis proposes that selection, therefore, favoured the evolution of a dynamic telomere structure, whose attrition tracks both the accumulation of oxidative damage to DNA (a risk factor for cancer inception) and cellular lineage proliferation (a biomarker of active cancer) and triggers irreversible cell-cycle arrest once excessive levels of either are reached, thereby blocking subsequent proliferation and yielding associated fitness benefits [27,29,35,67,84]. Consistent with this hypothesis, studies of diverse forms of human cancer have revealed that these aberrant cell lineages typically maintain their proliferative potential via mutations that activate or upregulate telomerase expression [67,85]. Accordingly, telomerase knock-out experiments in mice have been found to inhibit cancer inception and progression [86], fuelling interest in the therapeutic potential of anti-telomerase treatments in the fight against cancer [67]. Furthermore, comparative studies of mammals are suggestive of evolutionary changes in telomeric traits consistent with a function in tumour suppression: larger bodied species, whose larger number of cells are collectively expected to pose a greater cancer risk, tend to have somatic cells with shorter telomeres and lower levels of telomerase expression, which together may increase the stringency of telomere-mediated cancer surveillance ([27,67,74,84], see also [87] for supporting theory).

For cancer risk alone to account for the evolution of telomere attrition, however, the fitness benefit (over the life-course) from the attrition-mediated reduction in cancer risk would need to exceed any fitness costs that arise from telomere attrition. While this could well be the case, uncertainty regarding the prevalence, timing in the life-course and fitness consequences of cancer in natural populations leaves reason for caution when invoking a central role for cancer in selecting for telomere attrition. That said, several lines of evidence do collectively suggest that cancer has indeed been a significant selective force over evolutionary history [84,88–93]. First, cancer is a taxonomically widespread phenomenon, with rapidly accumulating evidence of its occurrence across the tree of life [88,89]. Second, while estimates of the incidence of cancer in animal populations suggest that it could be relatively rare (e.g. neoplasia was detected in 2.75% of autopsied captive mammals [92]), the fitness consequences of the detected subset of cancers would appear to be very high (e.g. 55% of the neoplasms detected in this study were considered the primary cause of death [92]). Moreover, such studies doubtless underestimate true cancer incidence (as acknowledged [92]), given the difficulty of exhaustively examining tissue and the potential for even microscopic neoplastic lesions to have fitness effects [89]. Advancing our understanding of the incidence and fitness consequences of cancer in natural animal populations is clearly a priority, but is likely to be hampered by cancer exerting its fitness effects in the wild principally via increases in susceptibility to other causes of mortality (such as predation and infectious disease), leading to the latter being logged as the cause of death [89]. Indeed, such survival costs associated with cryptic early-stage cancer phenomena could also readily account for the apparent rarity of advanced metastatic cancer in natural populations. Finally, a wealth of evidence suggests that diverse and elaborate anti-cancer mechanisms have evolved aside from telomere attrition, leaving further reason to implicate cancer as a potent selective force in natural populations [84,90,91,93]. Indeed, assessments made of the incidence and severity of cancer in the presence of such anti-cancer mechanisms must (by definition) underestimate the incidence or severity of cancer that originally selected for them.

Either way, if telomere attrition *per se* is minimally causal in current–future trade-offs and ageing then even minor fitness benefits arising from cancer mitigation could explain the evolution of telomere attrition despite the latter entailing minor costs to later-life performance. Recent evidence that cellular senescence in normal (non-cancerous) aged tissues *in vivo* may actually be triggered not by progressive telomere attrition but by telomeric DNA damage independent of telomere length [54,55] (see §2c(ii)) is of particular interest in this regard. While this finding reduces the plausibility of a strong causal role for telomere attrition *per se* in organismal ageing (see §2c(ii)), it need not undermine the hypothesis that cancer risk has selected for a telomere-mediated mechanism that *is* strongly causal in current–future trade-offs and ageing. This is because cancer risk may also have selected for this alternative pathway in which telomeric DNA damage triggers cellular senescence independent of telomere length [54,55].

#### (ii) The life-history regulation hypothesis

The biomedical literature has long recognized that progressive telomere attrition may allow the mounting of adaptive responses at the cellular level to DNA damage accumulation and excessive proliferation (see above). However, it would also seem plausible that telomere attrition (and/or the accumulation of telomeric DNA damage [54,55]), and the cell fates that they trigger, serve an additional function: allowing the adaptive regulation of organismal-level physiology, behaviour and life history in response to age-related declines in residual somatic integrity (the life-history regulation hypothesis). Adaptive life-history responses to organismal frailty have been predicted by theory and detected empirically, including impacts of advancing age or ill-health on reproductive investment (e.g. terminal investment [94]) and impacts of early-life adversity on life-history trajectories [1] and risk-taking (e.g. [95], see also [96]). The mechanisms that trigger these responses are poorly understood, and telomeres provide a credible but hitherto unexplored candidate for several reasons. First, telomere length (or the accumulation of telomeric DNA damage [54,55]) has the potential to act as a cue of both age- and disease-related frailty and past exposure to stress (see §2a). Second, in many species, telomere length is also predictive of mortality risk [36], which itself is likely to influence the profitability of alternative life-history trajectories. Third, multiple mechanisms exist whereby gene expression could be modulated according to telomere length in a potentially chromosome-specific manner and at great distance from the telomere in order to effect life-history regulation (e.g. telomere position effects and transcriptional signalling by telomeres [26,64,97]). Finally, there is growing evidence that telomeric traits predict various aspects of organismal behaviour [96].

The telomeric modulation of organismal-level function would presumably require (i) some form of averaging of telomere-dependent gene expression across a cell population (to eliminate problems arising from stochastic telomere attrition within individual cells [28]) and (ii) a means of this emergent signal modulating systemic signals to which the rest of the organism is exposed. Such a mechanism is plausible, given the similar processes that are achieved in biological clocks [98] and the potential for such a structure to yield systemic effects by interfacing, as clocks do, with the neuroendocrine system. Indeed, such a life-history pacemaker could also be based on the accumulation of senescent cells within a tissue, rendered mechanistically plausible by their distinctive secretory profiles [35]. Future studies should, therefore, consider the possibility that relationships between telomere length and some aspects of life history could be causal not because telomere-induced cellular senescence *disrupts* organismal function, but because telomere dynamics *adaptively regulate* organismal function.

## 4. Could telomere biology shed light on the evolution of life histories and ageing?

If telomere biology plays a proximate causal role in current–future trade-offs and ageing, could it shed new light on the evolution of life-history trade-offs and ageing? Whether telomere biology informs our understanding of the evolution of life histories and ageing trajectories hinges upon (i) the extent to which telomere biology sheds light on the evolutionary constraints that explain why selection has not decoupled life-history trade-offs (see §1) and (ii) the extent to which the evolution of telomeric mechanisms has influenced the ‘form’ of current–future trade-offs (i.e. the probability, magnitude and timing of the impacts of current actions on future performance) and ageing trajectories. I consider these two points in turn.

It is already clear that evolution proceeds subject to absolute constraints imposed by the laws of physics [1,2,20,23], and that these constraints alone (such as the need to allocate limited resources across competing traits) require the evolution of life-history trade-offs (see §1; [1,2,10,21]). However, the nature of the absolute constraints at play and the extent to which mechanistic constraints (again, those born of aspects of an organism's existing genetic, developmental and physiological mechanisms) are also important in life-history evolution remain a matter of debate (see §1; [9,12,20–23]). Attention to evolutionary explanations for telomeres being one proximate cause of current–future trade-offs (see §3) highlights (i) a potential role for diverse forms of constraint in the evolution of this mechanism and the trade-off that it is envisaged to yield, and hence (ii) scope for telomere research to shed light on the nature of these constraints. For example, progressive telomere attrition in somatic cells could be rendered adaptive by costs of telomere maintenance (the maintenance costs hypothesis), and these costs could arise solely from constraints on resource utilization (the absolute constraint already at the heart of resource allocation models of life-history evolution). However, other types of cost could also play a role (e.g. telomere maintenance could conceivably entail risks to chromosomal integrity) and be born instead of mechanistic constraints, absolute constraints or interactions between the two. Similarly, while a risk of cancer could also have selected for telomere attrition, cancer risk itself could be attributable principally to constraints on resource utilization (e.g. if cancer mitigation is too expensive to perfect [89–91,93]), but other forms of constraint could again be at play (e.g. if molecular proof-reading mechanisms are impossible to perfect [99,100]). If telomeres ultimately are found to play a causal role in current–future trade-offs, attempts to establish the importance of these various forms of constraint could, therefore, contribute significantly to our understanding of life-history evolution.

Telomere biology also has the potential to shed light on the ‘form’ of current–future trade-offs and ageing trajectories, given the potential for telomeric evolution to have modified these traits. With regard to impacts on the form of current–future trade-offs, imagine that in the absence of a telomere attrition strategy current investment in reproduction accelerated DNA damage accumulation (e.g. via increases in ROS production) and thereby increased cancer risk, yielding a particular form of current–future trade-off. The evolution of telomere attrition for the purposes of cancer surveillance in this scenario could then alter the nature of the cost of reproduction and the form of the current–future trade-off: effectively exchanging an elevated risk of stochastic organismal failure due to cancer for an acceleration of the gradual age-related decline in organismal performance (if telomere attrition was indeed causal in ageing). The extent to which the evolution of telomere attrition has modified ageing trajectories more broadly (independent of their deflection by current–future trade-offs) will depend upon a range of factors. Chief among these will be the extent to which telomere attrition *per se* is a proximate cause of ageing (see §2c(ii)) and whether telomere attrition affords age-specific pay-offs of similar magnitude in early and late life. For example, a strategy of telomere attrition could yield stronger age-specific net benefits (i) in early life, and hence exaggerate age-related declines in performance (e.g. if any net benefits arising from cancer mitigation are eroded at older ages by costs arising from senescent cell accumulation) or (ii) in late life, and hence ameliorate ageing trajectories (e.g. if in the absence of a telomere attrition strategy cancer risk would rise more acutely in late life). Insights into the nature and timing of the benefits and costs of a telomere attrition strategy might, therefore, ultimately clarify which of these scenarios applies.

Of particular interest, is the possibility that a strategy of telomere attrition is adaptive, but yields net *benefits* in early life and net *costs* in late life (see [35] for a similar suggestion regarding cellular senescence). This is important from an evolutionary perspective given the potential for relevant mutations to have the antagonistically pleiotropic effects envisaged in evolutionary explanations of ageing [5], but is of significant interest too from a biomedical perspective. The potential for late-life telomere elongation therapy to improve healthspan is presumably contingent upon continued telomere erosion in late-life being of net cost to late-life health; if telomere erosion was of net benefit throughout the life-course (e.g. via cancer mitigation effects at all ages) telomere elongation at any age could entail significant risks to health [60,101]. Predictions that telomere elongation therapy will improve natural healthspan [60,101] could, therefore, be strengthened by explaining why, if improving healthspan was that straightforward, selection had not already done so (e.g. by favouring the late-life upregulation of telomerase expression). One point of hope from a biomedical perspective is that the recent extraordinary rate of increase in human lifespan could well have outpaced the rate of evolutionary change in our late-life telomere biology, leaving scope for medicine to improve healthspan by addressing such a discord born of evolutionary lag.

## 5. Conclusion and future directions

The discussions above lead to several conclusions regarding our current understanding of the role of telomeres in the mechanisms and evolution of life-history trade-offs and ageing, each of which highlight outstanding areas of uncertainty that could profitably be the focus of future research. First, while it is mechanistically plausible that telomere dynamics are *one* proximate cause of current–future trade-offs and ageing in a subset of organisms, whether telomeres ever play a significant causal role in either phenomenon, relative to alternative mechanisms (such as oxidative damage to other biological structures), is currently far from clear (see §2). Of particular interest too is the possibility that telomeres do play a causal role but via telomere-length-independent mechanisms [54,55]: findings that (i) may require the reinterpretation of many findings previously attributed to effects of telomere attrition and (ii) lend strength to the view that telomere length *per se* may ultimately prove a non-causal biomarker of organismal somatic integrity. While experimental tests of the causality of such mechanisms are clearly needed, it is worth noting that attempts to exploit telomerase to test the causality of a role for telomere attrition *per se* (a growing area of interest in evolutionary ecology [62]) may be complicated in practice by the diversity of its actions aside from telomere elongation [26,52]. Given the expectation that declines in somatic integrity arise from complex interactions between multiple pathways [24,25], it may also ultimately prove difficult to distinguish no causal role for telomere attrition in organismal ageing from a minor causal role in which it acts in concert with a suite of other mechanisms. The field is also currently limited in the taxonomic generality of the mechanistic and evolutionary arguments that can be made, due to a primary focus to date on vertebrate model systems (principally birds and mammals) and the diversity in telomere biology already apparent both within and beyond these groups (e.g. [27,37]).

If telomeres do indeed play a proximate causal role in current–future trade-offs and ageing, it seems likely that such a mechanism would reflect an adaptive strategy. In this context, future research could usefully seek to distinguish two non-mutually-exclusive adaptive hypotheses, each of which could explain the evolution of a telomere-mediated current–future trade-off (and a role for telomeres in ageing) without the need to invoke the other: the maintenance costs hypothesis and the functional attrition hypothesis (see §3). Most discussions of adaptive explanations for telomere attrition invoke the functional attrition hypothesis, by highlighting that attrition may serve a function in cancer surveillance. However, it would be premature to focus exclusively on cancer as the likely selective agent in place of the simpler maintenance costs hypothesis, given uncertainty regarding the incidence and fitness consequences of cancer in natural populations and the potential for hitherto unexplored costs of telomere maintenance (see above). Key advances could, therefore, stem from research seeking to (i) clarify the mechanisms through which such maintenance costs could arise (which need not relate solely to resource allocation; see above) and (ii) provide critical tests of hypothesized functions for telomere attrition in natural populations (most critically, the cancer surveillance hypothesis). Given the possibility that telomere attrition also serves a function in life-history regulation (the life-history regulation hypothesis), future studies should also consider the possibility that relationships between telomere length and some aspects of life history could be causal *not* because telomere-induced cellular senescence disrupts organismal function, but because telomere dynamics are integral to mechanisms that adaptively regulate organismal function. Advances in our understanding of the selection pressures that have shaped any causal role for telomeres in life-history trade-offs and ageing ultimately have the potential to (i) shed new light on the nature of the evolutionary constraints at play in life-history evolution and (ii) help explain the form of the current–future trade-offs and ageing trajectories that we observe today.

## Data accessibility

This article has no additional data.
